# Redescription, molecular characterisation and taxonomic re-evaluation of a unique African monitor lizard haemogregarine *Karyolysus paradoxa* (Dias, 1954) n. comb. (Karyolysidae)

**DOI:** 10.1186/s13071-016-1600-8

**Published:** 2016-06-16

**Authors:** Courtney A. Cook, Edward C. Netherlands, Nico J. Smit

**Affiliations:** Unit for Environmental Sciences and Management, North-West University, Potchefstroom, South Africa; Laboratory of Aquatic Ecology, Evolution and Conservation, University of Leuven, 3000 Leuven, Belgium

**Keywords:** Haemogregarine taxonomy, *Hepatozoon*, Monitor lizard, Haematozoa, *Hemolivia*

## Abstract

**Background:**

Within the African monitor lizard family Varanidae, two haemogregarine genera have been reported. These comprise five species of *Hepatozoon* Miller, 1908 and a species of *Haemogregarina* Danilewsky, 1885. Even though other haemogregarine genera such as *Hemolivia* Petit, Landau, Baccam & Lainson, 1990 and *Karyolysus* Labbé, 1894 have been reported parasitising other lizard families, these have not been found infecting the Varanidae. The genus *Karyolysus* has to date been formally described and named only from lizards of the family Lacertidae and to the authors’ knowledge, this includes only nine species. Molecular characterisation using fragments of the 18S gene has only recently been completed for but two of these species. To date, three *Hepatozoon* species are known from southern African varanids, one of these *Hepatozoon paradoxa* (Dias, 1954) shares morphological characteristics alike to species of the family Karyolysidae. Thus, this study aimed to morphologically redescribe and characterise *H. paradoxa* molecularly, so as to determine its taxonomic placement.

**Methods:**

Specimens of *Varanus albigularis albigularis* Daudin, 1802 (Rock monitor) and *Varanus niloticus* (Linnaeus in Hasselquist, 1762) (Nile monitor) were collected from the Ndumo Game Reserve, South Africa. Upon capture animals were examined for haematophagous arthropods. Blood was collected, thin blood smears prepared, stained with Giemsa, screened and micrographs of parasites captured. Haemogregarine morphometric data were compared with the data for named haemogregarines of African varanids. Primer set HepF300 and HepR900 was employed to target a fragment of the 18S rRNA gene and resulting sequences compared with other known haemogregarine sequences selected from the GenBank database.

**Results:**

*Hepatozoon paradoxa* was identified infecting two out of eight (25 %) *V. a. albigularis* and a single (100 %) *V. niloticus* examined. Phylogenetic analyses revealed that *H. paradoxa* clustered with the ‘*Karyolysus*’ clade, and not with those of reptilian *Hepatozoon* spp.

**Conclusions:**

In addition to this being the first morphological and molecular characterisation of a haemogregarine within the African Varanidae, it is the first report of a species of *Karyolysus* infecting the monitor lizard family. Furthermore, this constitutes now only the third described and named *Karyolysus* species for which there is a nucleotide sequence available.

## Background

Within the apicomplexan order Adeleiorina, representatives of two haemogregarine genera, *Hepatozoon* Miller, 1908 and *Karyolysus* Labbé, 1894, are commonly reported infecting saurians. The genus *Hemolivia* Petit, Landau, Baccam & Lainson, 1990 on the contrary, even though reported parasitising saurian hosts, has but a single described species *Hemolivia mariae* Smallridge & Paperna, 1997 [1, 2]. Representatives of *Hepatozoon* are the most common and are cosmopolitan parasites found parasitising a wide range of vertebrate hosts from amphibians and reptiles to birds and mammals [3, 4]. *Karyolysus*, conversely, is known mainly as a saurian haemogregarine genus that primarily parasitises lizards of the family Lacertidae, but has also been reported from lizards of the Scincidae [1, 5–7]. Besides this discrepancy in vertebrate host preference of the species in the above haemogregarine genera, species in these genera also demonstrate different developmental patterns. Even though species of all three of the haemogregarine genera may be transmitted to the saurian host through the ingestion of the infected invertebrate vector, *Hepatozoon* spp. may be transmitted through a wide range of arthropod vectors (mosquitoes to ticks), whilst transmission of *Hemolivia* spp. and *Karyolysus* spp. has been recorded only through a tick and mite vector, respectively [1].

Whilst more than 30 *Hepatozoon* spp. have been recorded from saurians throughout Africa [8], *Karyolysus* spp. have mainly been reported from lacertid lizards of Europe and Asia [7]. Until Smith’s [3] revision of the Hepatozoidae, the genus *Karyolysus* comprised 11 species, Smith [3] reassigning two of these to *Hepatozoon*, now *Hepatozoon berestnewi* (Finkelstein, 1907) and *Hepatozoon bicapsulata* (Franca, 1910). To the authors’ knowledge, to date, nine species of *Karyolysus* are considered valid: *Karyolysus lacertae* Danilewsky, 1886; *Karyolysus lacazei* Labbé, 1894; *Karyolysus biretortus* Nicolle, 1904; *Karyolysus zuluetai* Reichenow, 1920; *Karyolysus subtilis* Ricci, 1954; *Karyolysus octocromosomi* Alvarez-Calvo, 1975; *Karyolysus latus* Svahn, 1975; *Karyolysus minor* Svahn, 1975; and the only species reported from sub-Saharan Africa *Karyolysus poleensis* Mutinga & Dipeolu, 1989 [3, 6, 7]. Descriptions of haemogregarine species were until recently based on morphological characteristics and life-cycle data [7]. This is particularly true of the haemogregarines described from saurians within southern Africa. Haemogregarines, specifically species of *Hepatozoon*, have been commonly recorded blood parasites of southern African saurians including those from lizard genera of the families Cordylidae and Varanidae [8, 9]. Within the African Varanidae, six species of *Hepatozoon* have been described, three of these from southern Africa (Table 1), the latter three comprising *Hepatozoon varani* (Laveran, 1905) from *Varanus niloticus* (Linnaeus, 1762) in South Africa [3, 6], *Hepatozoon camarai* (Dias, 1954) and *Hepatozoon paradoxa* (Dias, 1954) from *Varanus albigularis albigularis* Daudin, 1802 in Mozambique [3, 6].

**Table 1:** Species of haemogregarines of the genus *Hepatozoon* described from African varanids

Species	Type-host	Type-locality	Other hosts (localities)	Peripheral gamont/nucleus dimensions	Reference
*Hepatozoon borreli* (Nicolle & Comte, 1906) Smith, 1996^b^	*Varanus griseus* (Daudin, 1803)	Tunisia		7–8 × 2/1–3 × 1–2	[3]^b^, [6, 14]
*Hepatozoon camarai* (Dias, 1954) Smith, 1996^b^	*Varanus albigularis albigularis* (Daudin, 1802)	Mozambique		2 forms observed: banana-shaped: 11.75 × 5.00; long curved: 14.25–18.25 × 1.25–4.25/nucleus irregular	[3]^b^, [9]
*Hepatozoon paradoxa* (Dias, 1954) Smith, 1996^b^	*V. a. albigularis* ^1^	Mozambique	*V. niloticus* (Kenya)^2^; *V. a. albigularis* and *V. niloticus* (South Africa)^3^	6.75–7.50 × 4.25–5.50/nucleus not visible^1^; 8.1 × 5.2/nucleus irregular or not visible^2^; 6.99 × 4.39/nucleus not visible^3^	[9]^1^, [30]^2^, this study^3^, [3]^b^
*Haemogregarina roshdyi* Ramdan, Sauod, Mohammed & Fawzi, 1996 [probably *Hepatozoon roshdyi* (Ramdan, Sauod, Mohammed & Fawzi, 1996)]	*V. griseus*	Egypt		13–20 × 1.5–2.5/6.0–8.5 × 1.5–2.5	[40]
^*a*^ *Hepatozoon toddi* (Wolbach, 1914) Smith, 1996^b^	*Varanus niloticus* (Linnaeus, 1762)	The Gambia		10.3 × 2.5/not given	[3]^b^, [6, 15]
*Hepatozoon varani* (Laveran, 1905) Smith, 1996^b^	*V. niloticus* ^1^	South Africa	*V. niloticus* and *V. griseus* (Senegal)^2^; *V. niloticus* (?) (Portuguese Guinea)^3^; *V. niloticus* (The Gambia)^4^; *V. niloticus* (Senegal)^5^; *Varanus arenarius* Duméril & Bibron, 1836 (?) (French West Africa)^6^; *V. griseus* (French Sudan)^7^; *V. niloticus* (Kenya)^8^	14 × 3/not given^1^; 11–15 × 3/not given^2^; 12.0 × 13/3.5–4.5 × 3.0^3^; 10.3 × 2.5/not given^*a*4^; two forms: 12–14 × 2/not given, 10–12 × 4–5/5–6 × 4–5^5^; report only^6^; report only^7^; 12.7 × 4.6/not given^8^	[13]^1^, [41]^2^, [15]^3^, [42]^3^, [43]^4^, [44]^5^, [45]^6^, [46]^7^, [30]^8^, [3]^b^

^*a*^ *Hepatozoon* (syn. *Haemogregarina*) *toddi* (Wolbach, 1914) when first discovered was tentatively thought to be *Hepatozoon* (syn. *Haemogregarina*) *varani* (Laveran, 1905)

^b^Smith [3] during a systematic revision of species of the Hepatozoidae transferred many of the above species from the genus *Haemogregarina* Danilewsky, 1885 to the genus *Hepatozoon* Miller, 1908

^1–8^Corresponding description or report of the haemogregarine species and its bibliographic reference

The aim of the present study was thus to provide a morphological redescription of *H. paradoxa* and molecular data aiding in the correct taxonomic placement of this parasite.

## Methods

### Study area, *Varanus* spp. collection and blood preparation

Specimens of *Varanus albigularis albigularis* and *Varanus niloticus* were collected in daylight during the summer months of November 2013, February and November 2014, and February 2015 in the Ndumo Game Reserve (NGR) (26°52′00.0″S, 32°15′00.0″E), north-eastern KwaZulu-Natal (KZN), South Africa, bordering southern Mozambique [10]. Lizards were restrained by hand whilst blood and any haematophagous athropods were collected *in situ*. A small volume of blood (approximately one drop) was collected from the ventral caudal vein using an appropriately gauged (depending on the size of the lizard) sterile needle and 1 ml syringe. A small portion of the collected blood was used to prepare 2–3 duplicate thin blood smears and the remainder dropped into an equal volume of 70 % ethanol for future molecular analysis. Thin blood smears once air-dried in a dustproof container were fixed in absolute methanol and stained thereafter using a modified solution of Giemsa stain (FLUKA, Sigma-Aldrich, Steinheim, Germany) according to the methods of [11, 12].

### Screening of *Varanus* spp. blood smears

Smears were screened under a 100× oil immersion objective on a Nikon Eclipse E800 compound microscope (Nikon, Amsterdam, The Netherlands) and images were captured with an attached Nikon digital camera and accompanying software. Haemogregarines were identified to species level by comparing morphometric data to that of previous studies on African *Varanus* spp. haemogregarines [9, 13–15] (see Table 1). Parasitaemia was calculated per 100 erythrocytes, with *c.*10^4^ erythrocytes examined per blood smear [16–18].

### DNA extraction, PCR amplification and 18S rDNA sequence analysis

Ethanol-preserved blood samples were used for molecular work. Genomic DNA of haemogregarine species was extracted from the samples using a rapid DNA extraction method as detailed in the KAPA Express Extract Kit (Kapa Biosystems, Cape Town, South Africa). Based on previous studies, amplifying fragments of the 18S rRNA gene of reptile haemogregarines of the genera *Karyolysus* [7], *Hemolivia* [12] and *Hepatozoon* [19], identification of the parasite of the two *Varanus* species, two *V. a albigularis* and one *V. niloticus* (*n* = 3) from the current study was completed using the primer set HepF300 (5′-GTT TCT GAC CTA TCA GCT TTC GAC G-3′) and HepR900 (5′-CAA ATC TAA GAA TTT CAC CTC TGA C-3′). The PCR reactions were run targeting a fragment (approximately 600 nt) of the 18S rRNA gene [20]. Conditions for PCR were as follows: initial denaturation at 95 °C for 3 min, followed by 35 cycles, entailing a 95 °C denaturation for 30 s, annealing at 60 °C for 30 s with an end extension at 72 °C for 1 min, and following the cycles a final extension of 72 °C for 10 min as detailed according to previous methods [12, 18]. PCR reactions were performed with volumes of 25 μl, using 12.5 μl Thermo Scientific DreamTaq PCR master mix (2×) (2× DreamTaq buffer, 0.4 mM of each dNTP, and 4 mM MgCl_2_), 1.25 μl of each primer, and at least 25 ng DNA. The final reaction volume was made up with PCR-grade nuclease free water (Thermo Scientific, Vilnius, Lithuania). Reactions were undertaken in a Bio-Rad C1000 Touch™ Thermal Cycler PCR machine (Bio-Rad, Hemel Hempstead, UK). Resulting amplicons were visualized under ultraviolet light on a 1 % agarose gel stained with gel red using a Bio-Rad GelDoc™ XR+ imaging system (Bio-Rad, Hemel Hempstead, UK). Two PCR products from each sample were sent to a commercial sequencing company (Inqaba Biotechnical Industries (Pty) Ltd, Pretoria, South Africa) for purification and sequencing in both directions. Resultant sequences were assembled, and chromatogram-based contigs were generated and trimmed using Geneious Ver. 7.1 [21]. Sequences were identified using the Basic Local Alignment Search Tool (BLAST) [22], and deposited in the NCBI GenBank database under accession numbers KX011039 and KX011040.

Comparative sequences for species of *Hemolivia*, *Hepatozoon*, *Karyolysus*, *Haemogregarina*, *Dactylosoma* Labbé 1894 and *Babesiosoma* Jakowska & Nigrelli, 1956 parasitising reptiles, amphibians, mammals and ticks were downloaded from GenBank and aligned to the sequences generated in this study. *Adelina dimidiata* Schneider, 1875, *Adelina grylli* Butaeva, 1996 (GenBank: DQ096835–DQ096836) and *Klossia helicina* Schneider, 1875 (GenBank: HQ224955) were chosen as the outgroup to root the phylogeny.

Sequences were aligned using the MUSCLE alignment tool [23] implemented in Geneious 7.1. The alignment consisted of 47 sequences, manually trimmed to a total length of 968 nt. Uncorrected pair-wise distances (p-distance), base pair differences as well as parsimony informative sites and the number thereof were identified or determined with the MEGA6 bioinformatics software program [24] for the aligned 18S rDNA sequences between all available species appearing in the phylogenetic analyses.

To infer phylogenetic relationships of the aligned dataset both Bayesian inference (BI) and Maximum likelihood (ML) methods were used. A comprehensive model test was preformed to determine the most suitable nucleotide substitution model, according to the Akaike information criterion using jModelTest 2.1.7 [25, 26]. The best model identified was the Transversion Model plus with estimates of invariable sites and a discrete Gamma distribution (TVM+I+Γ). This model was substituted with the General Time Reversible model (GTR+I+Γ) for phylogenetic analysis, as this was the most appropriate model available with the best AICc score. The BI analysis was implemented from within Geneious 7.1 using MrBayes 3.2.2 [27]. The analysis was run twice over 10 million generations for the Markov Chains Monte Carlo (MCMC) algorithm. The Markov chain was sampled every 100 cycles, and the MCMC variant contained 4 chains with a temperature of 0.2. The log-likelihood values of the sample point were plotted against the generation time and the first 25 % of the trees were discarded as ‘burn-in’ with no ‘burn-in’ samples being retained. The ML analysis was performed using RAxML Ver. 8.1.22 [28] implemented in the raxmlGUI Ver. 1.3 [29]. The alpha-parameter selected was the GTR+I+Γ model, with support assessed using 1,000 rapid bootstrap inferences. Resulting trees were combined in a 50 % majority consensus tree.

### Ethics statement

This study received the relevant ethical approval (North-West University ethics approval no: NWU-00005-14-S3).

## Results

### Prevalence, parasitaemia and general observations of *H. paradoxa* in peripheral blood smears

**Fig. 1:**
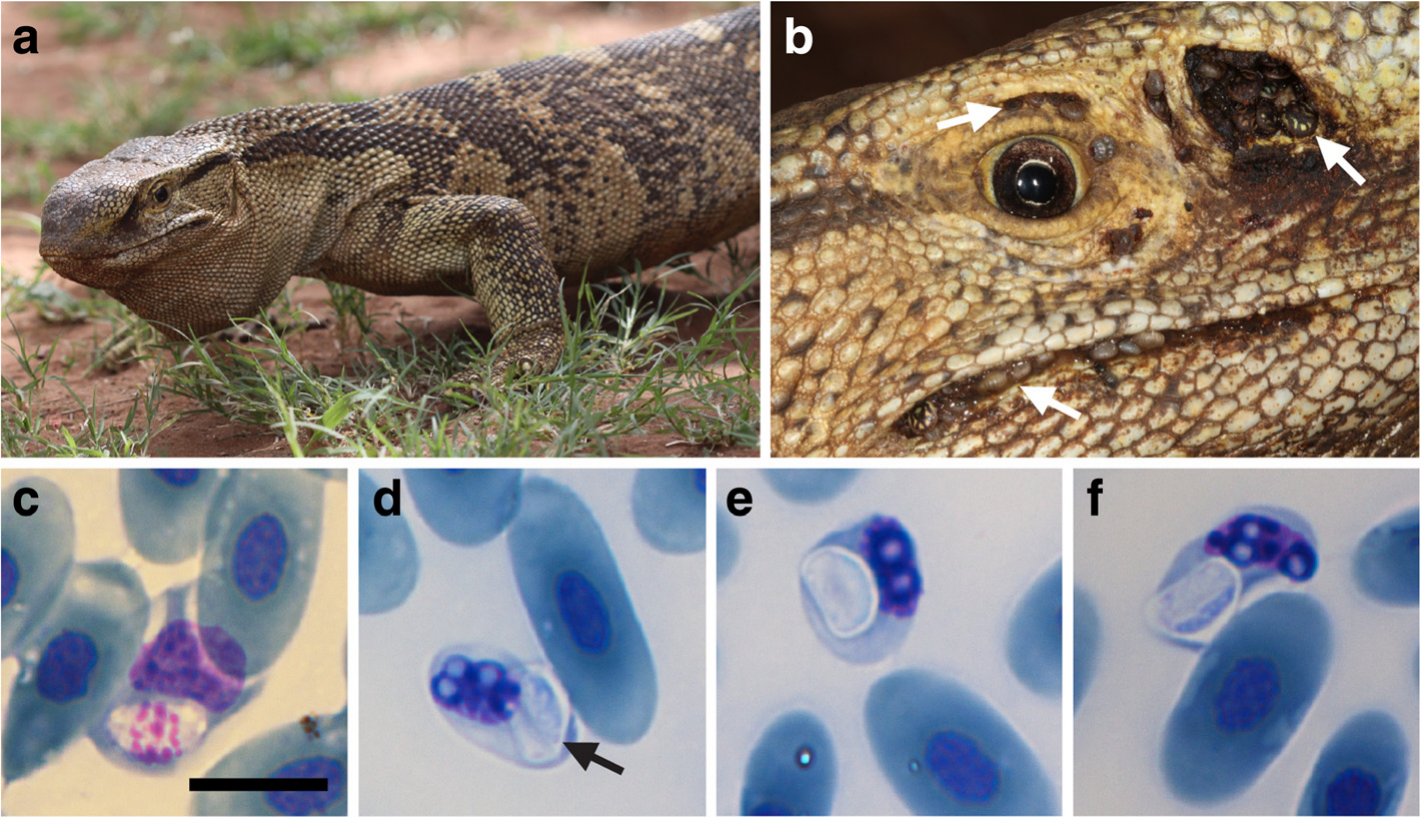
*Karyolysus paradoxa* (Dias, 1954) in varanid lizard *Varanus albigularis albigularis* Daudin, 1802. **a**–**b** *Varanus albigularis albigularis.* **b** Ticks of the species *Amblyomma exornatum* infesting the area above the eyes, the periphery of the mouth and deep into the nostrils (*arrows*). **c**–**f** Peripheral blood stages of *K. paradoxa* captured from the neohapantotype slide (NMB P 410). **c** Possible rare trophozoite stage, note that the young host erythrocyte cytoplasm and nucleus are still intact and that the parasite nucleus is visible and granular. **d**–**f** Mature gamonts within an erythrocyte in which shrinkage of the host cell is apparent and the nucleus destroyed resulting in a heavily vacuolated appearance. **d** Mature gamont in which folding of the gamont may be seen within the thick capsule (*arrow*). *Scale-bar*: 10 μm

**Fig. 2:**
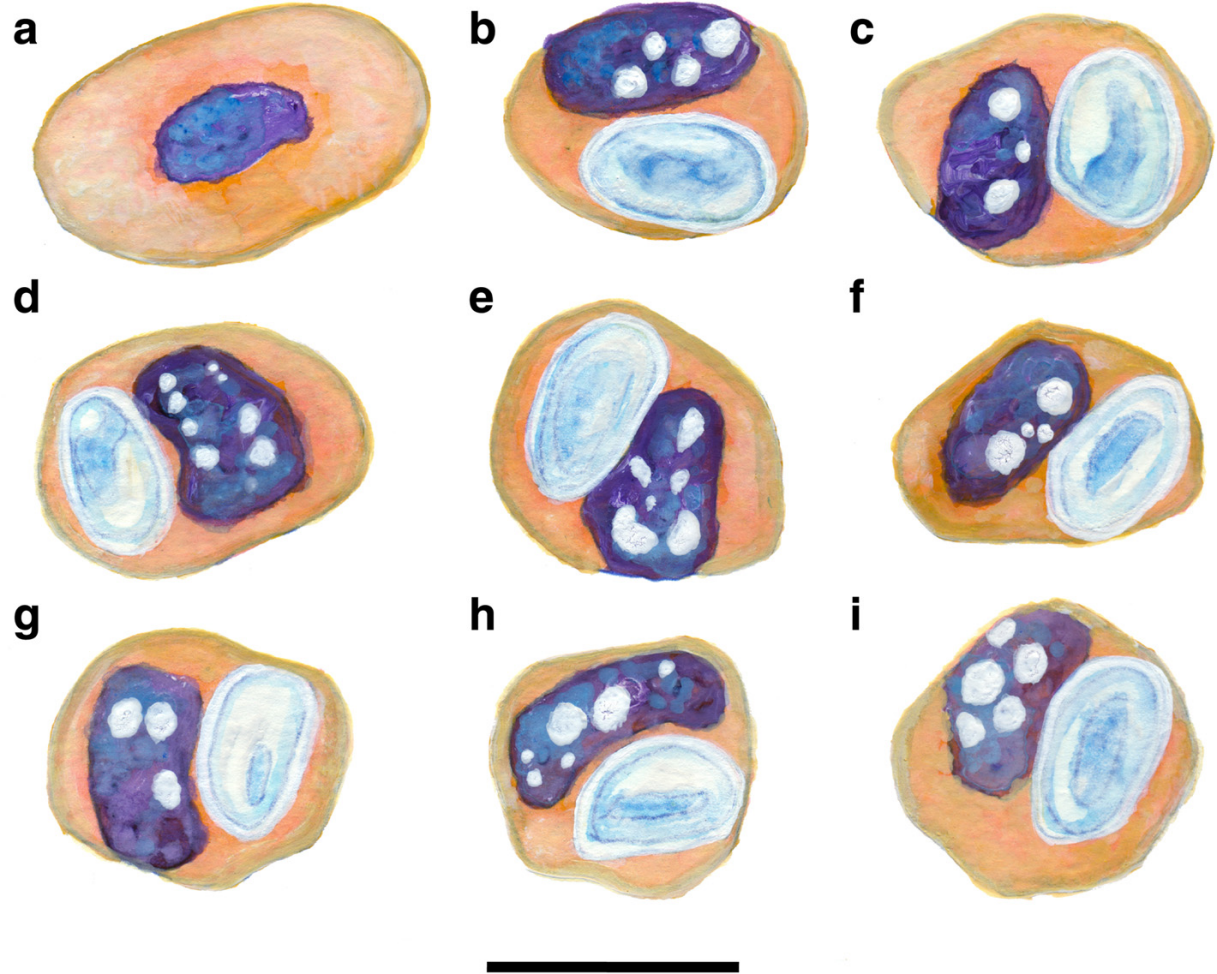
Illustration of *Haemogregarina paradoxa* Dias, 1954 in *Varanus albigularis albigularis* Daudin, 1802 [9]. **a**-**i** Redrawn and adapted from Dias (1954). Illustrations representing the original description of *Karyolysus paradoxa* (syns. *Hepatozoon paradoxa* and *Haemogregarina paradoxa*) ex *Varanus albigularis albigularis* from Mozambique. **a** Healthy non-parasitised erythrocyte. **b**-**i** Parasitised erythrocytes, note the shrinkage of the host cell, the heavy vacuolization of the host cell nucleus, and the thick capsule surrounding the gamont which results in the gamont nucleus being invisible. *Scale-bar*: 10 μm

Six adult and two juvenile *Varanus albigularis albigularis* (Fig. 1a, b) and one adult *Varanus niloticus* were captured, sampled for blood parasites and examined for possible haematophagous arthropods. The two juveniles were found to be negative for blood parasites, whilst 4/6 (67 %) adult *V. a. albigularis* and 1/1 (100 %) adult *V. niloticus* were found positive for haemogregarine infections. Two adult (33 %) (with a parasitaemia of *c*.5 and 20 %, respectively) *V. a. albigularis* and the *V. niloticus* (with a parasitaemia of *c*.0.2 %) were parasitised by a haemogregarine alike to *Hepatozoon paradoxa* described by Dias [9] (Figs. 1c–f and 2b–i). One of the two (17 %) *V. a. albigularis* (parasitaemia of 5 %) and the single *V. niloticus* were found to have a co-infection with another haemogregarine of a different *Hepatozoon* spp. (Cook, Netherlands & Smit, unpublished observations) (parasitaemia of *c.*0.4 and 3 %, respectively). The latter unidentified *Hepatozoon* species was also found infecting the remaining two parasitised *V. a. albigularis* (parasitaemia of *c*.0.1 and 0.5 %, respectively). All specimens of both species of *Varanus* were infested with adult and juvenile stages of the Leguan tick *Amblyomma exornatum* (Koch, 1844) (Fig. 1b), the two juvenile *V. a. albigularis* and *V. niloticus* demonstrating lower densities of these ectoparasites. These ectoparasites were found all over the animal, with the highest densities in the nostrils, often blocking them, also on the area surrounding the eyes, and along the edges of the mouth. It was not uncommon to find dead ticks and their remains deep within the nostril, their ability to exit blocked by later arrivals. Squashes of nymphal and adult female and male ticks provided no parasitic stages. No other haematophagous arthropods, including mites, were observed.

**Fig. 3:**
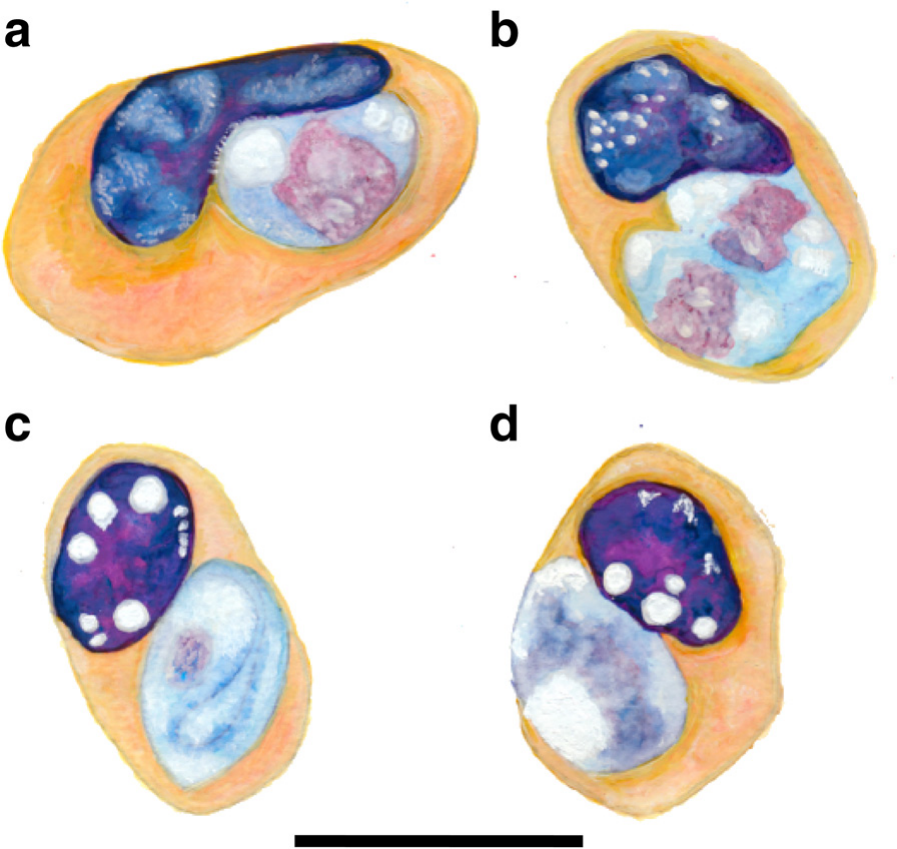
Illustration of an unknown haemogregarine by Ball (1967) [30] in *Varanus niloticus* (Linnaeus in Hasselquist, 1762). **a**–**d** Redrawn and adapted from Ball (1967) (his Figs. 19–22). Illustrations representing an unknown haemogregarine found as a concurrent infection to *Hepatozoon varani* (Laveran, 1905) (syn. *Haemogregarina varani*) in a *Varanus niloticus* from Kenya. **a** Possible trophozoite stage or young stage, shrinkage and vacuolation of the host cell and host cell nucleus not yet apparent. **b** Possible dividing stage. **c**–**d** Mature gamont stages, note the shrinkage and heavy vacuolation of the host cell erythrocyte. **c** Mature gamont folded within a capsule with possible nucleus. **d** Folding of the gamont and the gamont nucleus not observable through the capsule. *Scale-bar*: 10 μm (even though not provided by Ball, we have provided a scale matching to the in text description)

Stages of the *H. paradoxa*-like haemogregarine observed in peripheral blood smears from this study were compared morphologically with those observed in previous blood parasite studies of African varanids [9, 13–15] (Table 1). In size and morphology, the *H. paradoxa*-like stages observed during the current study conformed to those described by Dias [9] and Ball [30] (see Table 1, Figs. 1a–f, 2b–i and 3a–d, respectively). Two stages of the parasite were observed: a stage unreported by Dias [9], but possibly by Ball [30], a rare possible trophozoite stage (Figs. 1c and 3a), and what was identified as a mature intra-erythrocytic gamont stage (Figs. 1d–f, 2b–i and 3d, e).

### *Karyolysus paradoxa* (Dias, 1954) Cook, Netherlands & Smit, 2016

Syns *Haemogregarina paradoxa* Dias, 1954; *Hepatozoon paradoxa* Smith, 1996.

***Type-host*:***Varanus albigularis albigularis* Daudin, 1802, Squamata: Varanidae [9].

***Other hosts*:***Varanus niloticus* (Linnaeus in Hasselquist, 1762), Squamata: Varanidae [30]; present study.

***Vector*:** Unknown.

***Type-locality*:** Ndumo Game Reserve (26°54′18.5″S, 32°19′24.7″E), KwaZulu-Natal, South Africa (present study).

***Other localities*:** Maputo, Mozambique [9]. Ball [30] also described a haemogregarine morphologically alike to *K. paradoxa* in Marimonti, near Meru, Kenya.

***Type-material*:** Neohapantotype, 1× blood smear from the type-host *Varanus albigularis albigularis* and new designated locality (26°54′18.5″S, 32°19′24.7″E), deposited in the protozoan collection of the National Museum, Bloemfontein, South Africa under accession number NMB P 410. Other voucher material deposited that includes stages of *K. paradoxa*, 1× blood smear from *Varanus niloticus*, deposited in the protozoan collection of the National Museum, Bloemfontein, South Africa under accession number NMB P 411.

***Representative DNA sequences*:** Two sequences representing a 611 and 613 nt fragment of the 18S rRNA gene of *K. paradoxa* isolated from the type-host *Varanus albigularis albigularis*, deposited in the NCBI GenBank database under the accession numbers KX011039 and KX011040, respectively.

### Redescription (Figs. 1, 2 and 3)

***Trophozoite***. Rare, ovoid, with vacuolated cytoplasm, measuring 6.5–6.9 × 4.3–4.7 (6.7 × 4.5) μm (*n* = 2); nucleus with loose chromatin, staining pink (Fig. 1c). Both trophozoites parasitising young erythrocytes, no host cell distortion visible.

***Mature gamont.*** Rounded in shape, gamont seemingly folded within with a well-developed capsule (Fig. 1d–f), measuring 6.3–7.9 × 3.6–5.2 (7.0 × 4.4) μm (*n* = 20). Cytoplasm staining whitish-blue; nucleus not visible. Notable destruction of host cell cytoplasm and karyolysis of the host cell nucleus, causing an observable heavily vacuolated and foamy appearance (Fig. 1d–f).

### Remarks

The haemogregarine described in this study from South African *Varanus albigularis albigularis* and *Varanus niloticus* (Fig. 1d–f) was found to be morphologically similar to *Hepatozoon paradoxa* described by Dias [9] from a specimen of *V. a. albigularis* in neighbouring Mozambique (Fig. 2b–i). It shared a number of unique characteristics including destruction of the infected host erythrocyte, consisting of dehaemoglobinisation resulting in shrinkage of the host cell and destruction of the host cell nucleus (characteristic of a number of species of *Karyolysus* [1]) resulting in a heavily vacuolated appearance (Figs. 1d–f and 2b–i). Additionally, the haemogregarine in this study agrees well with the size of *H. paradoxa* in the original description of Dias [9] (mean 7.0 × 4.4 *vs* 7.0 × 4.9 μm) (Table 1).

The same unique characteristics were reported of a haemogregarine found infecting a *V. niloticus* by Ball [30] from Kenya, measuring on average 8.1 × 5.2 μm (Fig. 3c, d). However, in Ball’s [30] study, additional, presumably younger, stages were observed (similar to the young trophozoite stage found in our study) (Figs. 1c and 3a). Ball [30] also noted a single possibly dividing stage of these trophozoites (Fig. 3b). In cells parasitised by all these possible trophozoite stages, the host erythrocyte showed no shrinkage as of yet, but was according to Ball’s report abnormal in shape and staining. At first, Ball [30] did assume this parasite to represent younger stages of another haemogregarine that has been reported parasitising African varanids *Hepatozoon varani* (Laveran, 1905) Smith, 1996 (Table 1). However, based on the effects of the parasite resembling *H. paradoxa* as described by Dias [9], its destruction of the host cell and the host cell’s nucleus, he concluded that this parasite was not *H. varani*. Overall, for the *K. paradoxa* described in this study and the parasites described in the other two studies [9, 30], the nucleus and cytoplasm was not visible owing to what appeared to be a thick enclosing capsule as seen with the gamonts of species of *Hemolivia*, see [12, 31] (Figs. 1d–f, 2b–i and 3c, d). Only on rare occasion in the present study and that of Ball’s [30] was the parasite seen to be folding over on itself (Figs. 1d and 3c). Otherwise, the only evidence of this behaviour was a crescent shaped stain at the centre of the oval parasite (as seen in all three reports) (Figs. 1, 2 and 3). It is based on the above unique characteristics, particularly of the mature gamont stages, that we suggest all three reports are of the same parasite species *K. paradoxa*.

No hapantotype, according to the International Code of Zoological Nomenclature (ICZN) Article 73.3, was designated and deposited during the original description of *K. paradoxa* by Dias [9]. Ball [30] also did not identify the parasite to taxon level and did not deposit any voucher material. Furthermore, our efforts to locate any original specimens or voucher material were unsuccessful. In this study, *K. paradoxa* was collected from Ndumo Game Reserve, northern KwaZulu-Natal, South Africa, bordering the south of Mozambique. The original description by Dias [9] was collected in the vicinity of Maputo in the southern parts of Mozambique approximately 300 km from the NGR. Additionally, *K. paradoxa* in the present study was collected from the same host species *Varanus albigularis albigularis* as in the original description by Dias [9]. Based on the above, the mature gamont size comparisons, and the unique characteristics of the mature gamonts of *K. paradoxa* (destruction and shrinkage of the infected host erythrocyte, destruction of the host cell nucleus resulting in vacuolation, and the thick non-staining capsule) as described above for all three reports of this parasite, which includes the original description by Dias [9], and in accordance with ICZN Article 75.3, we designate a neohapantotype. The present study also includes both the description of an additional stage of the parasite (a trophozoite) and provides sequence data (fragment 18S rDNA), which was not provided by Dias [9] in his original description of this parasite species. This neohapantotype is deposited in the protozoan collection of the National Museum, Bloemfontein, South Africa under accession number NMB P 410.

### Sequence identification and phylogenetic analysis

**Fig. 4:**
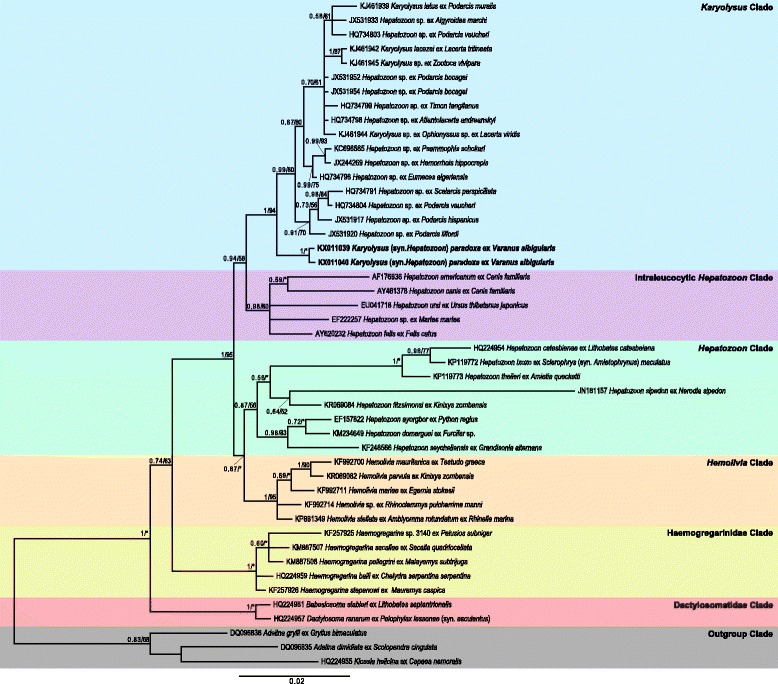
Phylogenetic analysis of *Karyolysus paradoxa* (Dias, 1954) based on 18S rDNA sequences. Bayesian inference (BI) and Maximum Likelihood (ML) analysis showing the phylogenetic relationships for two *Karyolyus paradoxa* isolates from the current study represented in bold, 17 *Karyolysus* and *Hepatozoon* species (representing the *Karyolysus* clade in *blue*), five mammal-infecting *Hepatozoon* species (representing the ‘intraleucocytic’ *Hepatozoon* clade in *purple*), eight herpatofaunal-infecting *Hepatozoon* species (representing the ‘intraerythrocytic’ *Hepatozoon* clade in *green*), five *Hemolivia* species (representing the *Hemolivia* clade in *orange*), five *Haemogregarina* species (representing the Haemogregarinidae clade in *yellow*), a *Babesiosoma* and *Dactylosoma* species (representing the Dactylosomatidae clade in *pink*) and two *Adelina* and one *Klossia* species (used as the outgroup in *grey*). All comparative sequences were downloaded from the GenBank database. Tree topologies for both the BI and ML trees were identical; the nodal support values (BI/ML) are represented on the BI tree

Amplicons of 611 and 613 nt for the 18S rRNA gene of *K. paradoxa* were obtained from the *V. a. albigularis* with the seemingly pure and highest parasitaemia infection using primer sets HepF300 and HepR900. No *K. paradoxa* isolate was obtained from the *V. niloticus*, likely due to its low parasitaemia in comparison to the concurrent infection of the other unidentified *Hepatozoon* species (unpublished data). The details of the species used in the phylogenetic analyses and presented in the consensus tree are provided in Table 2. The topology of both the BI and ML analyses were overall similar, with discrete monophyletic clades of known and likely belonging to *Karyolysus* species, *Hepatozoon* spp. of mammals (‘intraleucocytic’ *Hepatozoon* spp.), the herpatofauna (‘intraerythrocytic’ *Hepatozoon* spp.), *Hemolivia* spp., *Haemogregarina* spp., and the Dactylosomatidae (Fig. 4). Our results showed that *K. paradoxa* clustered within a major monophyletic clade containing both known (morphologically and ecologically confirmed) and likely belonging to *Karyolysus* species, and ‘intraleucocytic’ *Hepatozoon* species of mammals that were sister to the *Karyolysus* clade. This major clade was distinct from the major monophyletic clade containing the herpatofaunal ‘intraerythrocytic’ *Hepatozoon* spp. and *Hemolivia* spp.

**Table 2:** List of organisms used in the phylogenetic analyses of this study, with associated host, host family and host common name, GenBank accession numbers and references

Clade	Organism	Host	Family	Common name	Accession number	Reference
*Karyolysus*	*Karyolysus latus*	*Podarcis muralis*	Lacertidae	Common wall lizard	KJ461939	[7]
	*Hepatozoon* sp.	*Algyroides marchi*	Lacertidae	Spanish keeled lizard	JX531933	[34]
	*Hepatozoon* sp.	*Podarcis vaucheri*	Lacertidae	Andalusian wall lizard	HQ734803	[33]
	*Karyolysus lacazei*	*Lacerta trilineata*	Lacertidae	Balkan emerald lizard	KJ461942	[7]
	*Karyolysus* sp.	*Zootoca vivipara*	Lacertidae	Viviparous lizard	KJ461945	[7]
	*Hepatozoon* sp.	*Podarcis bocagei*	Lacertidae	Bocage’s wall lizard	JX531952	[34]
	*Hepatozoon* sp.	*Podarcis bocagei*	Lacertidae	Bocage’s wall lizard	JX531954	[34]
	*Hepatozoon* sp.	*Timon tangitanus*	Lacertidae	Moroccan eyed lizard	HQ734799	[33]
	*Hepatozoon* sp.	*Atlantolacerta andreanskyi*	Lacertidae	Andreansky’s lizard	HQ734798	[33]
	*Karyolysus* sp.	*Ophionyssus* sp. ex *Lacerta viridis*	Lacertidae	European green lizard	KJ461944	[7]
	*Hepatozoon* sp.	*Psammophis schokari*	Lamprophiidae	Schokari sand racer	KC696565	[47]
	*Hepatozoon* sp.	*Hemorrhois hippocrepis*	Colubridae	Horseshoe snake	JX244269	[35]
	*Hepatozoon* sp.	*Eumeces algeriensis*	Scincidae	Algerian skink	HQ734796	[33]
	*Hepatozoon* sp.	*Scelarcis perspicillata*	Lacertidae	Menorca wall lizard	HQ734791	[33]
	*Hepatozoon* sp.	*Podarcis vaucheri*	Lacertidae	Andalusian wall lizard	HQ734804	[33]
	*Hepatozoon* sp.	*Podarcis hispanicus*	Lacertidae	Iberian wall lizard	JX531917	[34]
	*Hepatozoon* sp.	*Podarcis lilfordi*	Lacertidae	Lilford’s wall lizard	JX531920	[34]
	*Karyolysus* (syn. *Hepatozoon*) *paradoxa*	*Varanus albigularis*	Varanidae	Rock monitor lizard	KX011039	This study
	*Karyolysus* (syn. *Hepatozoon*) *paradoxa*	*Varanus albigularis*	Varanidae	Rock monitor lizard	KX011040	This study
Intraleucocytic *Hepatozoon*	*Hepatozoon americanum*	*Canis familiaris*	Canidae	Domestic dog	AF176836	[48]
	*Hepatozoon canis*	*Canis familiaris*	Canidae	Domestic dog	AY461378	[49]
	*Hepatozoon ursi*	*Ursus thibetanus japonicus*	Ursidae	Japanese black bear	EU041718	[50]
	*Hepatozoon* sp.	*Martes martes*	Mustelidae	Pine marten	EF222257	[51]
	*Hepatozoon felis*	*Felis catus*	Felidae	Domestic cat	AY620232	[50]
*Hepatozoon*	*Hepatozoon catesbianae*	*Lithobates catesbeianus*	Ranidae	American bullfrog	HQ224954	[49]
	*Hepatozoon ixoxo*	*Sclerophrys* (syn. *Amietophrynus*) *maculatus*	Bufonidae	Flat-backed toad	KP119772	[52]
	*Hepatozoon theileri*	*Amietia quecketti*	Pyxicephalidae	Queckett’s river frog	KP119773	[18]
	*Hepatozoon sipedon*	*Nerodia sipedon*	Colubridae	Northern water snake	JN181157	[49]
	*Hepatozoon fitzsimonsi*	*Kinixys zombensis*	Testudinidae	Bell’s hingeback tortoise	KR069084	[12]
	*Hepatozoon ayorgbor*	*Python regius* (^a^ *Lamprophis* (syn. *Boaedon*) *fuliginosus*)	Pythonidae; ^a^Lamprophiidae	Ball python; brown house snake	EF157822	[53]
	*Hepatozoon domerguei*	*Furcifer* sp.	Chamaeleonidae	Chameleon species	KM234649	[54]
	*Hepatozoon seychellensis*	*Grandisonia alternans*	Indotyphlidae	Caecilian	KF246566	[55]
*Hemolivia*	*Hemolivia mauritanica*	*Testudo graeca*	Testudinidae	Mediterranean spur-thighed tortoise	KF992700	[36]
	*Hemolivia parvula*	*Kinixys zombensis*	Testudinidae	Bell’s hingeback tortoise	KR069082	[12]
	*Hemolivia mariae*	*Egernia stokesii*	Scincidae	Gidgee spiny-tailed skink	KF992711	[36]
	*Hemolivia* sp.	*Rhinoclemmys pulcherrima manni*	Geoemydidae	Painted wood turtle	KF992714	[36]
	*Hemolivia stellata*	*Amblyomma rotundatum ex Rhinella marina*	Bufonidae	Cane toad	KP881349	[37]
Haemogregarinidae	*Haemogregarina* sp._3140	*Pelusios subniger*	Pelomedusidae	East African black mud turtle	KF257925	[56]
	*Haemogregarina sacaliae*	*Sacalia quadriocellata*	Geoemydidae	Four-eyed turtle	KM887507	[57]
	*Haemogregarina pellegrini*	*Malayemys subtrijuga*	Geoemydidae	Snail-eating turtle	KM887508	[57]
	*Haemogregarina balli*	*Chelydra serpentina serpentina*	Chelydridae	Common snapping turtle	HQ224959	[49]
	*Haemogregarina stepanowi*	*Mauremys caspica*	Geoemydidae	Caspian turtle	KF257926	[56]
Dactylosomatidae	*Babesiosoma stableri*	*Lithobates septentrionalis*	Ranidae	Mink frog	HQ224961	[49]
	*Dactylosoma ranarum*	*Pelophylax lessonae* (syn. *esculentus*)	Ranidae	Pool frog	HQ224957	[49]
Outgroup	*Klossia helicina*	*Cepaea nemoralis*	Helicidae	Grove snail	HQ224955	[49]
	*Adelina dimidiata*	*Scolopendra cingulata*	Scolopendridae	Megarian banded centipede	DQ096835	[58]
	*Adelina grylli*	*Gryllus bimaculatus*	Gryllidae	Field cricket	DQ096836	[58]

^a^Experimental laboratory animal

The parasite collected in the present study that morphologically conformed to the original description of *Hepatozoon paradoxa* formed a sister taxon to the larger ‘*Karyolysus*’ clade (containing the known and likely belonging to *Karyolysus* species) in the phylogenetic analysis (Fig. 4). According to evolutionary divergence estimates the present material was most closely related to known *Karyolysus* spp. (at 97.7 %, p-distance = 0.02) than to species of the genera *Hepatozoon*, *Hemolivia* and *Haemogregarina* (Table 3).

**Table 3:** Representation of evolutionary divergence of the different clades in relation to *Karyolysus paradoxa* (Dias, 1954)

Clade	Similarity	p-distance	Nucleotide difference	Parsimony informative sites (%)
Known *Karyolysus* (4)	97.7	0.02	14 (11–18)	73
‘Intraleucocytic’ *Hepatozoon* (5)	95.7	0.04 (0.03–0.05)	26 (18–45)	29
*Hepatozoon* (8)	94.4	0.05 (0.03–0.09)	33 (21–56)	46
*Hemolivia* (5)	97.0	0.03	18 (17–20)	66
*Haemogregarina* (5)	94.7	0.05	32 (29–34)	68

Summative representation of clades shown in Fig. 4, showing clades with number of sequences in parentheses, the average percent similarity, average uncorrected p-distance with range in parentheses, average base pair difference with range in parentheses, and percent parsimony informative sites, all compared to the two sequences as shown in Fig. 4 of *Karyolysus* (syn. *Hepatozoon*) *paradoxa* from *Varanus albigularis albigularis*. Note that only the known *Karyolysus* species have been used from the *Karyolysus* clade

## Discussion

*Varanus albigularis albigularis* and *Varanus niloticus* are known to display somewhat different habitat preferences, preferring more terrestrial and aquatic environments respectively [32]. Both *V. a. albigularis* and *V. niloticus* can be found throughout South Africa from the more tropical Indian Ocean coastal belt in the East, west to the margins of the more arid Northern and Western Cape provinces [32]. Both species often occur sympatrically, particularly in Ndumo Game Reserve, rendering the finding of the same parasite in both these species not surprising.

As the morphological and developmental characteristics typical of *Haemogregarina* spp. had never been observed in haemogregarines of the herpatofauna, *K. paradoxa* was transferred along with many other species from the herpatofauna, birds and mammals, from the genus *Haemogregarina* (Haemogregarinidae) to the genus *Hepatozoon* (Hepatozoidae) by Smith [3] during a systematic review of the Hepatozoidae. However, during the first detailed revision of *K. paradoxa* provided here in the present study, morphologically this species shares more characteristics with members of the family Karyolysidae. *Karyolysus paradoxa* peripheral blood gamonts appear to be encapsulated as in the *Hemolivia* and destroy the host cell nucleus as in a number of species of *Karyolysus* (as mentioned above in Remarks) [1, 7]. However, it is imperative to take into account that these morphological features, specifically for the latter genus, are not always present [7].

The phylogenetic position of *K. paradoxa* was shown to be at the base of a clade containing undescribed species of *Hepatozoon*, many of these the results of molecular *Hepatozoon* spp. surveys [33, 34], and known species of *Karyolysus* [7]. This may suggest that these undescribed *Hepatozoon* spp. might rather be species of *Karyolysus*. The *Karyolysus* spp. clade is part of a larger clade including a sister clade of *Hepatozoon* spp. from mammals. This topology, as seen in this study, has been observed in a number of other studies [7, 33–37]. Karadjian et al. [37], in their attempt to understand the relationships of the different haemogregarine genera, particularly in respect to the conundrum of the polyphyletic *Hepatozoon* clade, proposed, based on their phylogenetic findings, that a number of *Hepatozoon* species (most of which have not been morphologically described) might rather represent species of *Karyolysus*. Given that it was only since the recent identification, description and molecular characterisation of *Karyolysus* spp. by [7], it is only now that we are beginning to realize this possibility. Moreover, it appears that the diversity of squamates parasitised by likely species of *Karyolysus* is fast increasing, a scenario which is likely only to intensify in future. A recent molecular *Hepatozoon* spp. survey by [38] shows haemogregarine isolates from geckos of the genus *Tarentola* Gray, 1825 (Phyllodactylidae) are also falling within what may be seen as the ‘*Karyolysus*’ clade; a clade that at present also includes haemogregarines isolated from species of the families Colubridae and Lamprophiidae (both snakes), Lacertidae and Varanidae (both lizards), and Scincidae (skinks).

Furthermore, the present study shows that *K. paradoxa* is most closely related to known *Karyolysus* species, followed by species of the ‘intraleucocytic’ *Hepatozoon* clade and then species of the ‘intraerythrocytic’ *Hepatozoon* and *Hemolivia* clades. *Karyolysus* and *Hemolivia* morphologically still belong within the same family (Karyolisidae), however, with the use of the 18S rRNA gene these two genera in this study and in others generally fall in different major clades. *Karyolysus* clusters in a major clade with the ‘intraleucocytic’ mammal *Hepatozoon*, whilst *Hemolivia* clusters in a major clade with the ‘intraerythrocytic’ herpatofaunal *Hepatozoon*; this finding is apparent in the present study, and also in [36, 37]. It is clear that the relationship between these two genera may possibly only be resolved by using a multi-gene approach as in [39].

## Conclusions

Based on the morphology and the molecular findings presented in this study, we recommend the following nomenclatural correction: *Karyolysus paradoxa* (Dias, 1954) (syn. *Hepatozoon paradoxa* (Dias, 1954) Smith, 1996, *Haemogregarina paradoxa* Dias, 1954) in the varanid lizards *Varanus albigularis albigularis* (type-host), and *Varanus niloticus*. Our results showed that *Karyolysus paradoxa* is as closely related to species within its current generic assignment in the ‘intraerythrocytic’ herpatofaunal *Hepatozoon* as it is with the more distantly related species of the *Haemogregarina*.

Besides this study representing the first morphological and molecular report of a haemogregarine within an African varanid, it is the first report of a species of *Karyolysus* infecting a host of the Varanidae. Furthermore, it represents the third described and named *Karyolysus* spp. for which there is a nucleotide sequence available. It is hoped that this study will encourage further molecular work on the Karyolysidae, particularly the genus *Karyolysus*.

This study also extends the host and distribution range of *K. paradoxa* from only a single specimen of *V. a. albigularis* in Mozambique to an additional two specimens in South Africa, as well as including *V. niloticus* as an additional host both in South Africa and Kenya. The distribution range of *K. paradoxa* falls within subtropical areas in South Africa, Mozambique and Kenya, and as such it would be interesting to see if this particular parasite is restricted to subtropical areas such as is the case with *Hemolivia parvula* (Dias, 1953) found parasitising *Kinixys zombensis* Hewitt, 1931 tortoises of South Africa and Mozambique; see [12], or if it is more widely distributed throughout different biomes as is the case with *Hepatozoon fitzsimonsi* (Dias, 1953) found parasitising several tortoise species in South Africa and Mozambique; see [19].

Even though tick squashes did not result in any observable parasitic stages future studies will focus on identifying possible vectors. Parasitic stages found in these possible vectors will be identified to species level based on both morphological and molecular findings.

With the conundrum of the larger *Hepatozoon* clade being polyphyletic and absorbing the *Hemolivia* and *Karyolysus*, it is important to increase the number of taxa from which we can work and ask deeper phylogenetic questions. However, besides the molecular characterisation of these species, it is still important to focus on their morphology and where possible attempt to elucidate their life-cycles in order to resolve the complex taxonomy of these organisms. More importantly, it is necessary to include another faster evolving gene or even mitochondrial genomes of these groups following [39] before we can make any well-informed decisions.
